# Lung function reduction and chronic respiratory symptoms among workers in the cement industry: a follow up study

**DOI:** 10.1186/1471-2466-11-50

**Published:** 2011-11-08

**Authors:** Zeyede K Zeleke, Bente E Moen, Magne Bråtveit

**Affiliations:** 1 Department of Public Health and Primary Health Care Occupational and Environmental Medicine University of Bergen, Kalfarveien 31, NO-5018 Bergen, Norway; 2 Centre for International Health, University of Bergen, Overlege Danielsens Hus, Årstadveien 21, NO-5020 Bergen, Norway; 3 Department of Occupational Medicine, Haukeland University Hospital, Norway

## Abstract

**Background:**

There are only a few follow-up studies of respiratory function among cement workers. The main aims of this study were to measure total dust exposure, to examine chronic respiratory symptoms and changes in lung function among cement factory workers and controls that were followed for one year.

**Methods:**

The study was conducted in two cement factories in Ethiopia. Totally, 262 personal measurements of total dust among 105 randomly selected workers were performed. Samples of total dust were collected on 37-mm cellulose acetate filters placed in closed faced Millipore-cassettes. Totally 127 workers; 56 cleaners, 44 cement production workers and 27 controls were randomly selected from two factories and examined for lung function and interviewed for chronic respiratory symptoms in 2009. Of these, 91 workers; 38 cement cleaners (mean age 32 years), 33 cement production workers (36 years) and 20 controls (38 years) were examined with the same measurements in 2010.

**Results:**

Total geometric mean dust exposure among cleaners was 432 mg/m^3^. The fraction of samples exceeding the Threshold Limit Value (TLV) of 10 mg/m^3 ^for the cleaners varied from 84-97% in the four departments. The levels were considerably lower among the production workers (GM = 8.2 mg/m^3^), but still 48% exceeded 10 mg/m^3^.

The prevalence of all the chronic respiratory symptoms among both cleaners and production workers was significantly higher than among the controls.

Forced Expiratory Volume in one second (FEV_1_) and FEV_1_/Forced Vital Capacity (FEV_1_/FVC) were significantly reduced from 2009 to 2010 among the cleaners (p < 0.002 and p < 0.004, respectively) and production workers (p < 0.05 and p < 0.02, respectively), but not among the controls.

**Conclusions:**

The high prevalence of chronic respiratory symptoms and reduction in lung function is probably associated with high cement dust exposure. Preventive measures are needed to reduce the dust exposure.

## Background

Cement is one of the most important building materials in the world. Exposure to cement dust has been demonstrated to have adverse effects on human health.

Several cross-sectional studies have reported reduction in lung function in workers exposed to high concentrations of cement plant dust [1-7]. The annual decrease in lung function has been calculated based on estimated cumulative dust exposure. In a cross-sectional study, Mwaiselage et al. [6] found an annual decline in FEV_1 _of 49.1 ml and FVC by 23.1 ml for an average worker exposed to total cumulative dust levels of 28.9 mg/m^3 ^year. Among never-smoking healthy adults, the expected age-related rate of decline in FEV_1 _range is 20-30 ml/year [8]. To identify excessive declines in FEV_1 _as soon as possible, annual measurements are preferable [8]. There are only a few follow-up studies of lung function among cement workers. Saric M et al. [9] found that the FEV_1_/FVC ratio measured on two occasions with an interval of four and eight years differed between cement and control workers. In that study, a significant reduction of FEV_1_, FVC and FEV_1_/FVC was found among the cement workers but not among the controls. Siracusa et al. [10] found a linear decline of FEV_1 _and FVC among cement workers who were checked in a follow-up study for 11 years. However, in that study the loss-to-follow-up was high (47.1%). Hence, more prospective studies are required to document yearly loss in lung function indices and changes in chronic respiratory symptoms among cement workers.

In a previous study from Ethiopia, the personal total dust exposure for cement cleaning workers was high (GM: 110.4 mg/m^3^). However, spirometry was not performed [11]. The main aims of the present study were to measure total dust exposure, to examine chronic respiratory symptoms and changes in FEV_1 _and FEV_1_/FVC among cement factory workers and controls that were followed for one year, and also to examine whether those having chronic respiratory symptoms were more prone to decreased lung function.

## Methods

### Study design and setting

**Figure 1:**
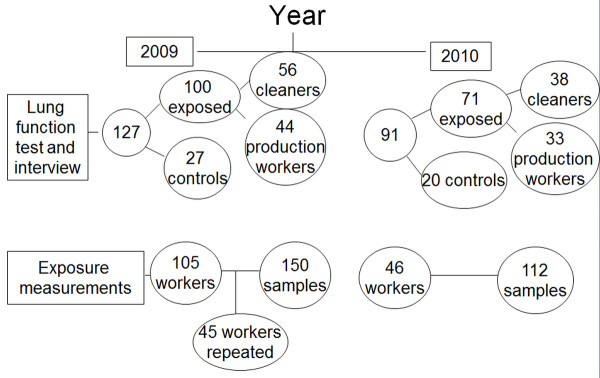
**Number of workers sampled for exposure measurements, lung function and interview in 2009 and 2010**.

This longitudinal study was conducted in two cement factories in Ethiopia which are described in our previous study (12). The total number of workers in the two factories in 2009 was 740 and 1336, respectively. In these factories, there were 117 cleaners, 181 production workers and 225 security workers. The baseline data for the present study were collected between May and August 2009, and comprised personal total dust measurement, spirometry and a questionnaire on respiratory symptoms. Similar examinations took place in 2010 at the same time of the year as in 2009. In 2009, 127 randomly selected workers were invited from the two factories and all of them were examined for lung function and interviewed for chronic respiratory symptoms. The participants comprised 56 cement cleaners, 44 cement production workers and 27 controls. Of these 91workers, 38 cement cleaners, 33 cement production workers and 20 controls were reexamined in 2010 with the same measurements (Figure 1). There were no interventions in these factories during the follow-up period.

### Exposed Workers

Cement dust-exposed workers from both plants were divided according to two main work tasks. The first group comprised cleaning workers and the second group included production workers. Cleaners clean leakages under and around the machines and conveyors using manual brooms, and they shovel piled dust back to the production line for reprocessing. They also assist maintenance workers when there is a large dust leakage due to the failure of machines. Production workers included operators and attendants who mainly visit the production line in order to monitor the process and ensure the smooth running of the machines in the respective departments. This category also included packers, loaders, dumper operators, dozer operators and belt attendants.

### Controls

Security workers from both factories served as a control group, since their dust exposure was considered to be low. The geometric mean of total dust exposure for the security workers from a previous study of an Ethiopian cement plant was 0.4 mg/m^3 ^(range: 0.18-0.9 mg/m^3^) [11].

### Exposure measurement

Lists of all production workers at the two factories were used to randomly select workers for dust sampling. One hundred fifty personal measurements of total dust among 105 selected workers were sampled in 2009; among these, 45 workers had two measurements each. One hundred twelve personal measurements of total dust among 46 workers were sampled in 2010 (1-2 measurements per worker for the production workers and 2-4 measurements per worker for the cleaners).

Personal total dust samples were collected on 37-mm cellulose acetate filters with pore size 0.8 μm placed in closed faced Millipore-cassettes situated in the breathing zone of the selected workers. The cassettes were attached to pumps (SKC Side Kick) at a flow rate of 2 l/min. The air flow was checked before and at the end of the sampling period using a rotameter. In 2009, the mean sampling time of total dust for the cleaners was 308 minutes (range:122-442 minutes), and for production workers, it was 333 minutes (180-450 minutes) during the eight-hour morning shift. Due to very high exposure levels in 2009, the sampling time in 2010 was reduced to 49 minutes (range: 22-100 minutes) for the cleaners, and 197 minutes (100-315 minutes) for production workers during the morning shift. The cement dust was measured quantitatively by gravimetric analysis on a microbalance scale (Mettler AT261), with a detection limit of 0.01 mg/m^3 ^in an ISO-certified laboratory (Eurofins, Denmark). In 2009, the fraction of total dust samples marked as overloaded were 68% [12]. In 2010, totally 48% of the total dust samples were marked as overloaded since loose dust was detected on the filter (60% and 24% total dust samples among cleaners and production workers, respectively).

We have used the Threshold Limit Value (TLV) of 10 mg/m^3 ^for inhalable particles not otherwise specified (PNOS) from American Conference of Governmental Industrial Hygienists, 2008 [13] as an occupational exposure limit.

Furthermore, ten measurements each for SO_2 _(5 in each plant) and NO_2 _(5 in each plant) near the kiln area were taken using Dräger tubes. The Dräger accuro pump was used to draw a calibrated 100 ml sample of air through the Dräger Tubes. The measuring ranges for the tubes were: 0.5-25 ppm for SO_2 _(Part No 6728491) and 0.5 - 25 ppm for NO_2 _(Part No CH30001), respectively. The samples were taken every other day for 5 days in each factory (Range: 24 to 30 hours between two consecutive measurements).

On the sampling days in 2010, notes on the weather conditions were taken, such as wind speed, humidity, temperature and rain fall data, from a wireless weather station (Classic Series WS 2029 LH) which was placed near the production area during the data collection period. There was no rain at any of the factories in the study during the fieldwork period; there was a moderate wind speed (range: 2.1-6.5 m/s), humidity was 19-70% and the outdoor temperature was between 20-37°C.

### Interview

A modified version of the British Medical Research Council (BMRC) questionnaire [14] was used for recording chronic respiratory symptoms. The questionnaire had three parts, which includes personal and work characteristics, smoking habits and chronic respiratory health symptoms. Using a standard translation procedure, the questionnaire was translated from English to Amharic and back to English. The questions on personal and work characteristics included age, educational level, employment history, previous illness, years worked in the cement factory and years worked in dusty industries elsewhere.

The study participants were asked if they had ever had illnesses like asthma, tuberculosis, chest injury/operation, abnormalities of the vertebral column/thoracic cage or any other severe debilitating disease such as a heart condition, diabetes mellitus, anemia or any neuromuscular disease. Those with any of these problems were excluded from the analysis. The chronic respiratory symptoms asked about were:

Do you usually cough first thing in the morning? 1. [Yes] 2. [No]

Do you usually cough during the day or at night? 1. [Yes] 2. [No]

Do you usually cough with sputum first thing in the morning? 1. [Yes] 2. [No]

Do you usually cough with sputum during the day or at night? 1. [Yes] 2. [No]

Are you troubled by shortness of breath when hurrying on level ground or walking up a slight hill? 1. [Yes] 2. [No]

Have you had attacks of wheezing in your chest at any time? 1. [Yes] 2. [No]

Do you usually experience chest tightness while at work or just after work? 1. [Yes] 2. [No]

Current smokers were those who smoked at the time of the study or who had stopped smoking less than one year ago. Ex-smokers were those who had quit at least one year before the survey. The workers were interviewed about the use of respiratory protective devices after their shifts. The same questionnaire was used during both data collection periods to document any changes in the respiratory health of the workers.

### Lung function test

A digital Spirare spirometer (SPS310) was used to measure the ventilatory function of the study subjects according to the American Thoracic Society (ATS) recommendations. The procedures for the ventilatory function test were explained individually to the workers. Spirometry was performed before the morning shift, while the workers were in a seated position. The pulmonary function profile included tests for FEV_1_, FVC, with a percentage ratio of FEV_1_/FVC. Spirometry was performed by the first author. The standing height and weight of the subjects were measured before the work shift in normal working clothes. The same spirometer and techniques were used in 2009 and 2010. Six spirometer recordings were excluded from analysis due to unacceptable readings.

### Data Analysis

SPSS Version 15 for Windows was used to analyze the data. The probability value of 0.05 and less was used as the criterion for statistical significance. Chi square test was used for categorical variables when comparing groups. A Wilcoxon signed-ranks test was used to analyze changes in chronic respiratory symptoms between baseline in 2009 and the follow-up. A dependent *t*-test was used to analyze changes in lung function indices during the one-year follow-up period. An independent *t*-test was used when analyzing mean differences between groups of workers. Analysis of variance (ANOVA) was also used for continuous variables. When this test produced significant results, post-hoc comparisons using the Bonferroni test were used to explore differences between each of the groups. Using the individual workers as a random factor, the within-worker (wwδ) and between-worker (bwδ) variance components of dust exposure (log_e_-transformed) were estimated using variance component structure in a linear mixed-effect regression model. Multiple linear regression was used to compare changes in lung function values between group of workers adjusting for age and height.

### Ethical Approval

The Regional Committee for Medical Research Ethics of Western Norway and the Regional Medical Research Committee in Oromia and the Mekelle Health Bureau of Ethiopia approved the study. The study design was explained to the managements of both factories. The nature of the studies was also explained to workers who were involved in the study and written consent was obtained from each participating worker both in 2009 and 2010.

## Results

### Exposure

The geometric mean total of dust exposure among cleaners was 432 mg/m^3^. The fraction of total dust samples exceeding the Threshold Limit Value (TLV) of 10 mg/m^3 ^for inhalable particles not otherwise specified (PNOS) [13] for the cleaners varied from 84-97% in the four departments (Table 1). The levels were considerably lower among the production workers (GM = 8.2 mg/m^3^), nevertheless, the geometric mean of 48% (range between departments 8-88%) exceeded the TLV.

**Table 1:** Personal total dust exposure (mg/m^3^) among cleaners and production workers in two cement factories in Ethiopia

Department									
	Work group	Ns	Nw	AM	GM	10-90^th ^percentile	wwδ	bwδ	**%>TLV of 10 mg/m**^**3**^
All	Cleaners	168	58	2215	432	12-6710	5.2	0.02	90.5
	Production workers	94	47	56	8.2	0.7-72	2.2	1.1	48
Crusher									
	Cleaners	44	16	2202	313	6-6600	7.0	0.0	84
	Production workers	24	12	5.2	2	0.6-19	1.3	0.0	8.3
Packing									
	Cleaners	31	11	1039	348	24-3280	3.2	0.0	97
	Production workers	33	18	35	26	7.7-76	0.6	0.05	88
Kiln									
	Cleaners	37	13	3310	419	8-10200	6.1	0.7	86.5
	Production workers	21	10	161	6	0.4-1130	5	0.4	24
Raw mill									
	Cleaners	56	18	2153	641	31-7160	3.8	0.1	94.6
	Production workers	16	7	37	9.2	0.5-147	2.2	2.2	56.3

*Notes: *Ns: Number of samples; Nw: Number of workers; GM: Geometric mean; AM: Arithmetic mean; wwδ: Within worker variance; bwδ: Between worker variance; Production workers: operators, belt attendants, kiln and cooler attendants, raw mill attendants, dumper operators, packers, loaders; % > TLV: Percentage of samples higher than the threshold limit value of 10 mg/m^3 ^for total dust

Among cleaners, the highest total dust exposure was in the raw mill department.

Comparing the four departments, the Bonferroni test indicated no significant differences in the log-transformed total dust levels for the cleaning workers. However, among production workers, there were significant differences in exposure levels between crusher and packing; as well as between the crusher and raw mill departments. The within-worker variance was also higher than the between-worker variance in both production workers and cleaners when stratified by section (Table 1).

The measurements by the Dräger tubes for SO_2 _(n = 10) and NO_2 _(n = 10) in both plants did not show detectable gas levels in the kiln area. Rain and wind speed did not affect the exposure variability in our study, as there was no rain during the sampling period and we found no correlation between wind speed and total dust exposure in any department. Only 21% of the exposed workers used respiratory protective devices. Those who did not use respiratory protective devices covered their mouths and noses with a piece of cloth.

### Cleaners, production workers and controls

The response rate for the interviews and spirometry for the invited workers was 100% and 71.1% in 2009 and 2010, respectively. According to the work task, the 2010 response rate was 68% for cleaners, 75% for production workers and 74% for controls.

The followed-up production workers and controls were not significantly different in age, smoking habits, education, height and weight except for employment years, where the production workers were employed for more years (11 years versus 6.7 years; p < 0.035).

However, the cleaners were significantly younger than the controls (32 years versus 38 years; p < 0.022). Cleaners and production workers were not significantly different in any other variable at baseline (Table 2.).

**Table 2:** Baseline characteristics of followed-up (FU) and lost to follow-up (LFU) workers according to work task in two cement factories in Ethiopia

	Cleaners (Cl)		Production workers (Pw)		Controls (C)		Significance level (FU)		
Variables	FU (n = 38)	LFU (n = 18)	FU (33)	LFU (11)	FU (20)	LFU (7)	Cl vs C	Cl vs Pw	Pw vs C
Age (years) ^***a***^	32 (10)	38 (9)*	36(10)	41(8)	39(10)	47(13)	**0.022**	0.109	0.320 ^c^
Height (m) ^***a***^	1.71(0.066)	1.72(0.069)	1.69(0.055)	1.69(0.046)	1.69(0.06)	1.72(0.08)	0.503	0.293	0.840 ^c^
Weight (kg) ^***a***^	61.8(8)	65.2(10.2)	64.3 (10.3)	60.2(7.9)	61.2(9)	62.7(9.8)	0.798	0.253	0.270 ^c^
Employment (years) ^***a***^	9(6.1)	13(7.6)*	11(7.3)	14(8)	6.7(7)	12.7(8.9)	0.237	0.157	**0.035 **^c^
Current smokers ^***b***^	2(5.3)	0(0)	1(3)	0(0)	0(0)	0(0)	0.296	0.641	0.432 ^d^
Non smokers ^*b*^	36(94.7)	18(100)	35(97.2)	11(100)	20(100)	7(100)	0.703	0.510	0.678 ^d^
Ex-smokers ^***b***^	2(5.3)	0(0)	5(15.2)	4(36.4)	1(5)	0(0)	0.966	0.238	0.258 ^d^
Primary education only ^***b***^	12(31.6)	12(66.7)*	9(27.3)	4(36.4)	5(25)	4(57.1)	0.601	0.692	0.856 ^d^
Protective device ^***b***^	7(18.4)	0(0)	8(24.2)	0(0)	0(0)	0(0)	**0.041**	0.549	**0.017 **^d^

**^a ^**= arithmetic mean (standard deviation); **^b ^**= number (%); ^C ^= Independent t-test, ^d ^= Chi square test,

### Loss to follow-up versus followed-up

The followed-up and loss-to-follow-up workers were not significantly different in smoking habits, height, weight, and use of respiratory protective devices at baseline. However, the followed-up workers were younger, worked fewer years, and were less educated than those who were loss to follow-up, and these differences were significant among the cleaners (Table 2). The loss to follow-up workers had a slightly higher prevalence than the followed-up workers; cough (62 vs. 48%), chest tightness (41 vs. 30%) and wheezing (27 vs. 20%), but the differences were not significant.

### Chronic respiratory symptoms

In 2009, cleaners had a significantly higher prevalence of morning cough, shortness of breath and chest tightness than controls (p < 0.001). Though not significant, they also had a higher prevalence of wheezing at baseline (Table 3).

**Table 3:** Prevalence of chronic respiratory symptoms among exposed and control workers in two cement factories in 2009 and 2010

	2009			2010			Significance level p 2009 Vs 2010		
Symptoms	Cleaners (n = 38)	Pw (n = 33)	Controls (n = 20)	Cleaners (n = 38)	Pw (n = 33)	Controls (n = 20)	Cleaners	Pw	controls
	n (%)	n (%)	n (%)	n (%)	n (%)	n (%)			
Morning cough	28(73.7)	13(39.4)	3(15)	18(47.4)	11(33.3)	2(10)	**0.008**	0.317	0.655
Cough day/night	22(57.9)	9(27.3)	2(10)	17(44.7)	11(33.3)	1(5)	0.166	0.414	0.564
Cough with sputum	28(73.7)	14(42.4)	3(15)	16(42.1)	10(30.3)	2(10)	**0.003**	0.102	0.655
Cough with sputum, day/night	21(55.3)	5(15.2)	2(10)	15(39.5)	10(30.3)	1(5)	0.083	**0.025**	0.564
Shortness of breath, when hurrying	27(71.1)	15(45.5)	2(10)	21(55.3)	14(42.4)	2(10)	0.109	0.763	1.000
Shortness of breath, when walking	4(10.5)	4(12.1)	0(0)	2(5.3)	0(0)	0(0)	0.414	**0.046**	1.000
Stop for breath	4(10.5)	2(6.1)	0(0)	1(2.6)	0(0)	0(0)	0.180	0.157	1.000
Wheezing	9(23.7)	8(24.2)	1(5)	13(34.2)	7(21.2)	0(0)	0.248	0.739	0.317
Chest tightness	17(44.7)	9(27.3)	1(5)	15(39.5)	12(36.4)	2(10)	0.564	0.257	0.564

Pw = Production workers, Wilcoxon signed ranks test

The production workers had a significantly higher prevalence of shortness of breath (p < 0.005) and chest tightness (p < 0.008) compared to the controls in 2009. They also had a higher prevalence of cough and wheezing, but not significantly so. In 2009, the cleaners had significantly higher prevalence than production workers for cough (p < 0.001) and shortness of breath (p < 0.012). When cleaners and production workers were merged, the prevalence of all chronic respiratory symptoms among this group (exposed) was significantly higher than among the controls.

Very few workers in the control group reported chronic respiratory symptoms at baseline and at follow-up (Table 3).

All the chronic respiratory symptoms among the cleaners and as well as among the production workers were higher than among the controls in 2010 (Table 3). Among the controls, the prevalence of chronic respiratory symptoms did not differ between baseline and follow-up. Among cleaners, the prevalence of morning cough was significantly higher in 2009 when compared to 2010. Among production workers, instances of cough with sputum, day/night increased significantly from 2009 to 2010. However, shortness of breath when walking had a reduced prevalence in 2010 when compared to 2009.

### Lung function

At baseline in 2009, FEV_1_value among cleaners was slightly higher when compared to the production workers and controls (Table 4). However, the differences in FEV_1_, FVC, and FEV_1_/FVC between cleaners and controls were not significant, even though the mean age of the cleaners was 7 years younger than controls. Furthermore, no significant differences in lung function were found between production workers and controls, or between cleaners and production workers.

**Table 4:** Lung function among workers in two cement plants and among controls at baseline (2009) and at follow-up (2010)

Lung function		Cleaners		Production workers		Controls	
Indices		(n = 38)	p*	(n = 33)	p*	(n = 20)	p*
FEV_1 _(L/min)	2009	3.46(0.67)		3.23(0.55)		3.33(0.76)	
	2010	3.36(0.65)		3.19(0.57)		3.30(0.70)	
ΔFEV_1_		-0.099(0.18)	0.002	- 0.092(0.26)	0.05	-0.032(0.27)	0.61
FVC (L)	2009	4.05(0.69)		3.82(0.58)		3.84(0.77)	
	2010	4.01(0.65)		3.79(0.53		3.80(0.72)	
Δ FVC		-0.038(0.31)	0.45	- 0.027(0.30)	0.60	-0.041(0.36)	0.62
FEV_1_/FVC	2009	85.19(6.3)		84.17(5.96)		86.32(4.34)	
	2010	83.49(7.5)		82.36(6.58)		85.83(6.01)	
ΔFEV_1_/FVC		-1.70(3.41)	0.004	-1.81(4.40)	0.02	- 0.485(3.52)	0.55

*Paired t-test

The followed-up and loss-to-follow-up workers were not significantly different in FEV_1_, FVC, and FEV_1_/FVC in any work categories at baseline, 2009 (data not shown).

FEV_1 _and FEV_1_/FVC were significantly reduced from 2009 to 2010 among the cleaners and production workers, but not among the controls (Table 4). FVC did not change significantly in any work category. The one-year reduction in FEV_1 _among cleaners, production workers and controls was 99 ml, 92 ml and 32 ml, respectively. When cleaners and production workers were merged, FEV_1 _and FEV_1_/FVC were significantly reduced from 2009 to 2010 in this group (exposed), but not among the controls. The mean changes in FEV_1 _and FEV_1_/FVC among cleaners and production workers were greater than for the controls, but not significantly (Table 4). These changes were still not significant after adjusting for age, height, smoking and employment years in a multiple linear regression analysis.

Cleaners who reported chronic respiratory symptoms at baseline, such as morning cough or shortness of breath, had reduced FEV_1 _and FEV_1_/FVC in the follow-up period compared to those who did not have these symptoms (Table 5). This was not found among production workers.

**Table 5:** Mean baseline values and mean changes in lung function during the follow-up period stratified by the presence of chronic respiratory symptoms.

Lung function	Baseline values	Changes	p*	Baseline values	Changes	p*
Indices	Mean (SD)	Mean (SD)		Mean (SD)	Mean (SD)	
**Cleaners**						
	**With morning cough n = 28**			**Without morning cough n = 10**		
FEV_1 _(L/min)	3.49(0.73)	-0.12(0.19)	0.003	3.37(0.46)	-0.047(0.15)	0.344
FVC (L)	4.05(0.73)	-0.034(0.34)	0.603	4.05(0.59)	-0.049(0.19)	0.450
FEV_1_/FVC	85.76(6.56)	-2.18(3.79)	0.005	83.59(5.65)	-0.34(1.38)	0.458
	**With shortness of breath n = 27**			**Without shortness of breath n = 11**		
FEV_1_	3.53(0.71)	-0.08(0.16)	0.016	3.27(0.55)	-0.14(0.22)	0.056
FVC	4.09(0.70)	-0.04(0.21)	0.292	3.94(0.67)	-0.02(0.48)	0.879
FEV_1_/FVC	85.92(5.73)	-1.13(1.79)	0.003	83.4(7.61)	-3.08(5.64)	0.100
**Production workers**						
	**With morning cough n = 13**			**Without morning cough n = 20**		
FEV_1_	3.21(0.46)	-0.013(0.25)	0.848	3.24(0.61)	-0.023(0.25)	0.722
FVC	3.88(0.47)	-0.063(0.31)	0.490	3.78(0.65)	-0.004(0.29)	0.946
FEV_1_/FVC	83.18(5.13)	0.39(2.26)	0.544	84.82(6.49)	-0.25(4.88)	0.381
	**With shortness of breath n = 15**			**Without shortness of breath n = 18**		
FEV_1_	3.36(0.55)	-0.09(0.23)	0.155	3.12(0.55)	-0.093(0.28)	0.184
FVC	3.84(0.50)	-0.016(0.17)	0.734	3.80(0.65)	-0.037(0.37)	0.682
FEV_1_/FVC	85.95(5.04)	-0.913(2.88)	0.240	82.69(6.4)	-2.55(5.31)	0.057

* paired t-test

## Discussion

The cement factory workers, when compared to controls, had a higher prevalence of chronic respiratory symptoms and a significant reduction in lung function in the follow-up period of one year.

The total dust exposure among the production workers in our present study is similar to the total dust levels for cement production workers in Malaysia (GM: 8.52 mg/m^3^) [2] but is higher than the levels found in the USA (AM: 7.5 mg/m^3^) [15] and in Norway (AM: 7.4 mg/m^3^) [16]. However, the measured total dust level among cleaners was very high, and even higher than for cement cleaning workers in our previous study from another cement factory Ethiopia (GM: 110.4 mg/m^3^) [11]. In developed countries, cement industries use more efficient dust control methods, such as enclosure of dust emitting machinery, general mechanical ventilation in the production areas, wet dust suppression during cleaning activities and use of local exhaust ventilation from the crusher and packing machinery [17]. Such control methods were lacking in the cement factories investigated in this study. Furthermore, in our present study, cleaning is accomplished exclusively by sweeping with dry manual brooms while shoveling is executed with shovels. The fraction of total dust samples exceeding 10 mg/m^3 ^in our study was 91% for cleaners and 48% among production workers, which is higher than for total dust samples in a Tanzanian cement plant, where 39% exceeded the TLV [18]. The within-worker variance was higher than the between-worker variance in both job categories in the present study. For the cleaners, this is due to the varying fraction of time spent on cleaning and working under or close to dust emitting machineries from day to day [12]. Generally, the time spent on outdoor activities and the mobility among production workers have been reported to be associated with high day-to-day (within-worker) variability [19,20] and may also contribute to the high within-worker variability in the present study.

The total dust levels in the present study might be underestimated due to the detection of loose dust on 68% and 48% of the dust samples in 2009 and 2010, respectively. However, both dust captured in the filter and the loose dust was measured (12). Despite the reduced sampling time in 2010, the overloading could not be totally avoided. Hence, a more precise estimate of the sampling time could have been performed to reduce the uncertainty during the gravimetric analysis of the filters. It might be questioned whether the relative short sampling time in 2010 reflects exposure levels that are representative for the 8 hour shift for the particular workers. However, for the selected workers, the dust samples were taken at random time periods during the 8 hour shift, i.e. 1-4 samples per worker, although not more than one sample per day per worker. Thus, we have assumed that the random selection of sampling periods results in representative exposure for the respective groups of workers.

As there was no improvement carried out to reduce the dust level in the factories during the follow-up period, the high values of total dust levels found in our study poses an increased risk of workers developing respiratory disorders. Only 21% of the exposed workers used respiratory protective devices while the rest covered their mouths and noses with a piece of cloth, which is probably not effective in protecting them from dust exposure.

In both 2009 and in 2010, cleaners and production workers had significantly more chronic respiratory symptoms than the controls. These effects are probably associated with the high concentrations of dust in the working environment. Our SO_2 _and NO_2 _measurements did not show detectable levels, indicating that the cleaners and production workers were exposed to low concentrations of these irritating gases.

Even though the cleaners were younger, they had the highest prevalence of respiratory symptoms. The prevalence of respiratory symptoms in general is assumed to increase with age, [21] thus supporting our suggestion that there is an association between cement dust exposure and chronic respiratory symptoms. Due to the low number of workers with respiratory symptoms among the controls, we did not perform logistic regression analysis to adjust for confounders. The high prevalence of chronic respiratory symptoms for the production workers in our study is in agreement with Mengesha and Bekele [3]. In a study of three Ethiopian factories, researchers found a higher prevalence of chronic respiratory symptoms among cement and yarn workers than among cigarette workers. Noor [2] also found increased prevalence of chronic respiratory symptoms among cement workers exposed to increased levels of dust. Our findings also confirm results from other previous cross-sectional studies reporting a higher prevalence of respiratory symptoms among exposed cement workers when compared with controls [1,4,5]. Comparing symptom prevalence between studies is difficult because there are several methodological differences. In previous cross-sectional studies many factors vary, such as the study population, dust concentration, duration of employment, age, smoking habits and how the respiratory symptoms are defined. Smoking can be a confounder in the development of respiratory symptoms in the cement industry [22]. In the present study, only two cleaners and one production workers were smokers and therefore, this factor is not important.

The cleaners had a significantly higher prevalence of cough than the production workers at baseline. Increased prevalence of cough may be due to high dust exposure among cleaners caused by resuspension of dust particles during the shoveling of piled dust that may produce a continuous supply of dust to the breathing zone. In our previous study, [12] the fraction of total to respirable dust was considerably higher among cleaners than among production workers. Thus, for the cleaners, a considerably larger proportion of the dust by mass is expected to be deposited in the upper part of the airways than is the case for the production workers.

Some symptoms were lower at follow-up and we have no explanation for this. A similar finding was also reported in a 5-year follow-up study among employees in Norwegian smelters, where they found decreases in symptoms such as cough and wheezing during the follow-up periods [23].

Despite the short follow-up period of one year, we found that FEV_1 _and FEV_1_/FVC were significantly reduced from 2009 to 2010 among the cleaners and production workers but not among the controls. The "true" decrease in lung function might be even more pronounced than what we found since a learning effect might be present in repeated lung function measurements [24]. Five years of follow-up is recommended to more reliably estimate an individual's rate of FEV_1 _decline. However, to identify excessive declines as soon as possible, annual measurements are preferable [8]. Our finding was in agreement with Saric M et al. [9] who found that the decline in FEV_1_, FVC and FEV_1_/FVC was larger among the cement workers than the controls after adjusting for age, previous cement exposure, symptoms of chronic bronchitis, smoking and re-examination interval. However, the examination interval in that study was four and eight years. For the control workers, the present study is in agreement with Hnizdo et al. [8] who reported a 20-30 ml/year expected decline in FEV_1 _among never-smoking healthy adults. Mwaiselage et al. [6] found a decline of 49.1 ml in FEV_1 _and 23.1 ml in FVC annually for a cement worker who is 38 years old, a non-smoker, and 170 cm tall, exposed to a total cumulative dust level of 28.9 mg/m^3 ^year. The decline in FEV_1 _in our present study is almost double, and for the cleaners, the dust level was much higher than this. In an eleven-year longitudinal study, Siracusa et al. [10] found a decline of FEV_1 _and FVC among cement workers who were non-smokers or light smokers (< 1.25 pack-years at the date of first employment). However, there was a substantial loss-to-follow-up (47.1%), and the loss to follow-up had lower lung function values than those who were followed up. In our study, the response rate at baseline was very high, and the loss to follow-up rate was lower (29%) than in previous studies. This high response rate might be due to highly motivated workers, since no such study had been performed in these factories before. However, the lost workers had worked more years and were older than the followed up workers among the exposed groups. Thus, a healthy worker effect can not be excluded. However, due to low employment rates in Ethiopia, this might have less impact on the results than in other countries, since workers might continue working even though they fall ill.

Cleaners who reported chronic respiratory symptoms such as cough and shortness of breath at baseline had reduced FEV_1 _and FEV_1_/FVC respectively, compared to those who did not report these symptoms. This finding was in agreement with Saric et al. [8] who found that in the group of healthy workers, the initial values of ventilatory indices were significantly higher than in workers with chronic bronchitics. Our findings suggest that workers with respiratory symptoms may be prone to a reduction of lung function related to excessive dust exposure.

One weakness of the present study is that the follow-up period is short. However, we found significant decreases in FEV_1 _and FEV_1_/FVC among both cleaners and production workers; even though it is known that the variability in FEV_1 _is high after a follow-up period of only one year [8]. Another weakness of the present study is that no tests were performed on infectious diseases such as tuberculosis and HIV. However, the control groups were from the same place and we have no reason to conclude that the findings can be explained by any epidemic of infection. The study population in the present study is relatively small and recruited from only two cement industries. However, these two factories are the largest in Ethiopia in terms of production capacity. The results of this study might be generalized for the working environment in similar plants with the same work routines in Ethiopia and East Africa. This might also be the case in some of the cement plants world-wide.

## Conclusions

The high prevalence of chronic respiratory symptoms and reduction in lung function is probably associated with cement dust exposure. Preventive measures are needed to reduce the dust exposure.

## Competing interests

The authors declare that they have no competing interests.

## Authors' contributions

ZKZ designed and conducted the study, undertook the analysis, made revisions to the manuscript after consultation with the other authors. BEM and MB participated on the design and analysis, conducted review and provided scientific support throughout the project and comments on the manuscript. All authors have read and approved the final manuscript.

## Pre-publication history

The pre-publication history for this paper can be accessed here:

http://www.biomedcentral.com/1471-2466/11/50/prepub
